# Government, governance, and place-based approaches: lessons from and for public policy

**DOI:** 10.1186/s12961-023-01074-7

**Published:** 2023-11-29

**Authors:** Bojana Klepac, Amy Mowle, Therese Riley, Melinda Craike

**Affiliations:** 1 Mitchell Institute for Education and Health Policy, Victoria University, Footscray Park Campus, Pathways in Place, Melbourne, VIC Australia; 2 Institute for Health and Sport, Victoria University, Melbourne, VIC Australia

**Keywords:** Public policy, Public health, Place-based approach, Document analysis, Policy analysis, Government

## Abstract

**Background:**

Place-based approaches are increasingly applied to address the determinants of health, many of which are complex problems, to ultimately improve population health outcomes. Through public policy, government actions can affect the effectiveness of place-based approaches by influencing the conceptualisation, development, implementation, governance, and/or evaluation of place-based approaches. Despite the important role of public policy, there has been limited examination of public policy related to place-based approaches. We add to the limited knowledge base by analysing Australian national public policy, to explore: (1) the definitions, conceptualisations, and characteristics of place-based approaches in public policy; (2) the government’s perception and communication of its role in place-based approaches; and (3) the extent to which government policy reflects the necessary conditions for successful place-based governance developed by Marsh and colleagues, namely localised context, embedded learning, and reciprocal accountability.

**Methods:**

This research was underpinned by the Theory of Systems Change and methodologically informed by the READ approach to document analysis. Ritchie and Spencer’s framework method was utilised to analyse the data.

**Results:**

We identified and reviewed 67 policy documents. In terms of conceptualisation, common characteristics of place-based approaches related to collaboration, including community in decision-making, responsiveness to community needs, and suitability of place-based approaches to address complex problems and socio-economic determinants of health. Three roles of government were identified: funder, partner, and creator of a supportive policy environment. From the three criteria for successful place-based governance, *localised* *context* was the most dominant across the documents and *reciprocal* *accountability* the least.

**Conclusions:**

Based on our findings, we drew key implications for public policy and research. There was a disproportionate emphasis on the bottom-up approach across the documents, which presents the risk of diminishing government interest in place-based approaches, potentially burdening communities experiencing disadvantage beyond their capacities. Governments engaged in place-based approaches should work towards a more balanced hybrid approach to place-based approaches that maintain the central functions of government while allowing for successful place-based governance. This could be achieved by promoting consistency in conceptualisations of ‘place-based’, employing an active role in trust building, advancing the creation of a supportive policy environment, and embedding ‘learning’ across place-based approaches.

**Supplementary Information:**

The online version contains supplementary material available at 10.1186/s12961-023-01074-7.

## Introduction

It is widely acknowledged that public policy plays a crucial role in reducing health inequities and inequalities, and enhancing population health outcomes [1–4]. The role of public policy in reducing health inequities has continued to gain prominence following the 1978 Declaration of Alma-Ata and the 1986 Ottawa Charter for Health Promotion. The Declaration of Alma-Ata underscored the influence of non-medical “causes of causes”, that is social (e.g., housing), economic (e.g., education, employment), and environmental determinants of health [5], highlighting the need for intersectoral efforts to address inequities. The Ottawa Charter for Health Promotion emphasised government responsibility in mitigating ‘health gaps’, and stipulating ‘healthy public policy’ as a principal action area [6]. These foundational multi-national commitments facilitated dialogue between sectors and the related but often-siloed public health domains—research, practice, and public policy [6, 7]. They also laid the groundwork for the Health in All Policies approach, which draws attention to the need for government action outside the health sector encouraging the systematic consideration of health implications across sectors to improve population health outcomes [8].

Since the Health in All Policies approach was introduced, related ideas and practices such as “joined-up government” or “whole of government” approaches [9, 10], “collaborative governance” [11], “networked governance” [12], “policy integration” [13], and horizontal and vertical coordination between and across sectors [10, 14] have continued to inform public health. Although these ideas and practices have some differences, they all aim to enhance the effectiveness of public policies and public administration in addressing the determinants of health, many of which are complex problems requiring cross-sectoral and collaborative action [15, 16]. Consistent with the practices of cross-sectoral and collaborative action, place-based approaches have gained traction in public policy.

### Association between place and health

The importance of *place* [17–24] is driven by the acknowledgement that health disparities are ‘unevenly geographically distributed’ between areas experiencing relative disadvantage and those of prosperity [25–29]. This ‘hot policy topic’ ([30], p. 562) has led to efforts to incorporate the concept of *place* into strategies and actions designed to improve population health outcomes for those living in areas of entrenched disadvantage. Recognising that health outcomes differ according to where people live or place, governments increasingly look to place-based approaches to address health disparities [18, 31, 32]. There are differing ways in which *place* can be conceptualised, (i.e., place can encompass different dimensions such as physical, social, cultural, and digital) to inform public policy. Place-based approaches often conceptualise *place* as the geographic site of locational (dis)advantage, focusing on the contexts and circumstances of “people in place”, with public policies focused on improving these circumstances [33]. Consistent with this conceptualisation, in this study, place is used to denote a focus on the interrelationships between contexts, activities, communities and cultures tied to specific geographic location [34].

Given this conceptualisation of place, place-based approaches focus on understanding and realigning local systems with the needs of the target population to address complex problems [35, 36]. This is compatible with and complements systems thinking perspectives. Systems thinking perspectives conceptualise a *place* as a complex system, with inter-connected components, characterised by feedback loops, emergent properties, non-linearity and adaptation. Systems thinking methodologies can be applied to understand the components and properties of local systems and are therefore useful for those who wish to implement and support place-based approaches [37].

### The role of government in place-based approaches

High-income countries, such as Australia, Canada, New Zealand, the United Kingdom, and the United States, have a history of developing, trialling and implementing place-based approaches [18, 19, 38, 39]. Governments across all levels often shape the conceptualisation, governance structures, development, implementation, and evaluation of place-based approaches through public policy, funding streams and other forms of government engagement. Public policies frame the understandings and expectations of place-based approaches by establishing what is ‘normative and expected, sanctioned or rewarded’ ([40], p. 208) within any given political system. It follows that the ‘influences and consequences’ of government actions can substantially impact the success (or lack thereof) of place-based approaches [14, 41].

Despite the important role of governments in place-based approaches, there have been few attempts to systematically analyse public policies related to place-based approaches [32, 42] Further, limited research has investigated how governments perceive place-based approaches and their role within them. Given the role of public policy in reducing health inequalities and inequities, the relative lack of research exploring the role of public policy in place-based approaches is a notable gap. One way this gap can be addressed is through analysis ‘about or on’ and ‘for’ [43] public policy on place-based approaches.

### Rationale for analysis of public policies related to place-based approaches

Analysis of government policy related to place-based approaches is important for a range of reasons. First, there are concerns that the use of the term *place-based* *approach* may become a “catchall” used by governments to describe ‘an array of potentially inconsistent policy agendas’ that do not reflect a consistent understanding of what a place-based approach implies [44–46]. An analysis of government perspectives on and conceptualisation of place-based approaches can: 1. surface potential inconsistencies across public policies; 2. aid in the development of a set of common features of place-based approaches within and across public policy; and 3. inform a degree of conceptual clarity that enhances development, implementation, and evaluation of place-based approaches.

Second, the significance of good governance, encompassing the structures, processes, and relationships that shape decision-making within a group, has been recognised as critical for place-based approaches [44, 47, 48]. Although *governance* has a variety of meanings across a rich body of literature, a common thread implies an evolution of governing practices in which the distinctions ‘between and within the public and private sectors have become blurred’ ([49], p. 17). The general turn from *government* to *governance* indicates a recognition that tackling complex problems requires a shift from traditional top-down approaches to the active involvement of non-state actors (e.g., community members, practitioners, researchers) [49–51]. A focus on governance is central to place-based approaches, which often attempt to improve population health outcomes through mechanisms of collaborative governance, that is, working with diverse stakeholders to create sustainable change in the places where people live [20, 52]. Despite the absence of a clear consensus on what constitutes effective governance in place-based approaches, Marsh and colleagues propose that 'localised context,' 'embedded learning,' and 'reciprocal accountability' [48] are essential conditions for successful governance. Given the pivotal role of government in governance of place-based approaches, it is timely to examine whether public policies related to place-based approaches incorporate principles that foster successful governance.

### Aims of the paper

Through an analysis of Australian national (i.e., federal level) public policy, this paper aims to explore:

1. the definitions, conceptualisations, and characteristics of place-based approaches in public policy;
2. the government’s perception and communication of its role in place-based approaches; and
3. the extent to which government public policy reflects the necessary conditions for successful place-based governance [48].

By building conceptual clarity around place-based approaches from a public policy perspective and developing an understanding of governments’ roles in place-based approaches, this paper will enhance the future development, implementation, evaluation, governance, and communication of public policy related to place-based approaches and inform a research agenda on place-based approaches concerning governance and public policy. This paper will also inform advocacy, practice, and policy efforts by individuals and organisations engaged in place-based approaches and other approaches embodying practices such as “networked governance”, “joined-up government” and “collaborative governance”.

## Methodological approach

### Study context: Pathways in Place program

This study is a part of broader program of research called *Pathways* *in* *Place:* *Co-Creating* *Community* *Capabilities* (Pathways in Place), funded by the Paul Ramsay Foundation. The program is focused on advancing the science and practice of place-based systems change approaches and co-led by two Australian universities—Victoria University, Victoria and Griffith University, Queensland.

This study was conducted by the Pathways in Place-Victoria University team (www.pathwaysinplace.com.au/victoria-university). The work of the Pathways in Place-Victoria University and this study are underpinned by our *Theory* *of* *Systems* *Change* [53], which proposes that an enabling public policy environment is needed to support the development and implementation of place-based approaches and capacity-building for place-based systems change. Within different program work streams, we work with diverse stakeholders, including practitioners, policymakers, and community members, to build capacity for effective place-based systems change approaches. This analysis was a foundational piece of work for the program, designed to inform our future work with policymakers. Although we have discussed this work with policymakers, we did not formally involve them in the study.

### Overarching approach

This research was methodologically informed by the READ approach to document analysis [54], and applied Ritchie and Spencer’s framework method [55] to analyse the data. In addition, we applied systems thinking as a sensitising lens to ensure we pay attention to the interrelationship of public policy and place.

#### Search strategy

The search was conducted by two authors (BK and AM) from 15th April to 1st May 2022. The primary search was performed through the government departments’ websites, which were directly queried for all mentions of ‘place-based’ or ‘place based’ using in-built search functions. We deliberately used an inclusive search to identify and include all relevant (both health and not health-related) government documents on place-based approaches. Focusing on population health outcomes, we are interested in how place-based approaches were used in relation to the wider determinants of health. As many determinants of health lay outside the traditional jurisdiction of health departments, our review included all departments. By not limiting our search to health-related policy documents, we captured the breadth of place-based policies that could potentially impact health. For the primary search, the stopping conditions were met when all relevant documents from each search had been extracted (i.e., ‘evidence exhaustion’) [56].

The websites of all 14 departments of the Australian Federal Government [57] were included in the primary search, namely: the Department of Agriculture, Water and the Environment; the Attorney-General’s Department; the Department of Defence; the Department of Education, Skills, and Employment; the Department of Finance; the Department of Foreign Affairs and Trade; the Department of Health; the Department of Home Affairs; the Department of Industry, Science, Energy and Resources; the Department of Infrastructure, Transport, Regional Development and Communications; the Department of the Prime Minister and Cabinet; the Department of Social Services; the Department of the Treasury; and the Department of Veterans’ Affairs.^1^ The names and structures of the Commonwealth government departments are liable to change in the post-electoral period of the electoral cycle. For simplicity, we have used the department names and structures that were current at the time of data collection.

To complement the primary search, a secondary search was conducted of the websites associated with each department using Google’s site search function. This function allows the user to search any indexed site or document for the specified search terms within a specific domain (e.g., the Department of Social Services website was queried with the search term *site:dss.gov.au* *place-based).* There is no established guidance on the number of results that should be screened in Google but previous research reported screening the first 100 results (see [58]). Because this was a complementary search, we made a pragmatic decision to screen the first 50 results for each query.

#### Inclusion criteria and document selection

As recommended in the READ approach, we determined the specific parameters around search strategy and inclusion and exclusion criteria based on the study's aims.

To be included, the document had to meet three selection criteria:

1. the document is one of the following: policy, framework, guideline, plan (e.g., strategic plan, implementation plan, corporate plan etc.), strategy, statement by a minister or a secretary, media release, discussion paper, or report (e.g., annual reports);
2. the document is publicly accessible online; and
3. the document is explicitly developed, enacted, published, or authored by one or more of the Australian federal Government departments.

Meeting notes, event descriptions, reports, and documents commissioned by the department(s), documents that contained a disclaimer such as ‘the opinions, comments and/or analysis expressed in this document are those of the authors and do not necessarily represent the views of the Department of…’, grant applications/calls for funding, and presentation materials (e.g., Power Point presentations) were excluded.

We applied no specific date or timeframe for the document’s inclusion/exclusion. A total of 181 documents containing the term ‘place-based’ or ‘place based’ were identified. Two authors (BK and AM) then conducted an independent screening process that included the manual exclusion of duplicates and the screening of full texts for inclusion based on pre-defined eligibility criteria. Following this process, a total of 67 documents were included in the study. Any discrepancies regarding the selection of documents were resolved through an open discussion between two authors (BK and AM), and the final selection of documents was finalised in May 2022.

#### Data extraction

For each of the 67 documents identified, we extracted the following data into an Excel spreadsheet: title, author(s), department(s), year of publication/last update, document type (e.g., action plan, annual report, media release), whether place-based approach was defined, the definition of place-based approach, other mentions of place-based, and alternate terms used to describe place-based. We then divided the documents into primary (*n* = 19) and secondary (*n* = 48). The primary documents were purposefully and independently selected by two authors (BK and AM) for in-depth analysis. Documents were classified as ‘primary’ if they contained enough relevant information about place-based approaches to warrant an in-depth analysis. Secondary documents were retained to provide broader insight into the definitions and conceptualisations of place-based approaches across the documents and highlight temporal trends related to using the term 'place-based' across different departments. Additionally, they were utilised to provide supporting information around context or further insight on broader trends, patterns and perspectives during the in-depth analysis of primary documents. For example, two annual reports [59, 60] were analysed in-depth as primary documents. Given the seven-year gap between the publication of these annual reports, we drew on mentions of place-based approaches in the annual reports published from 2015 to 2018 [61–63], to provide additional context to the analysis. Discrepancies between authors in document categorisation were resolved through an open discussion.

#### Data analysis

In this step, we used the framework method [55] to analyse the primary documents. The five key stages involved in the analysis were: 1. familiarisation with the data; 2. coding; 3. applying the analytical framework; 4. charting data into the framework matrix; and 5. mapping and interpretation of the data [55, 64]. The first stage of the analytical process involved gaining a deep familiarisation with the data, in which each primary document was carefully examined. The documents were imported into the online version of qualitative analysis software *ATLAS.ti* [65] and independently coded by two authors (BK and AM; second stage). Consistent with the framework method [55], we drew upon ‘a priori issues,’ ‘emergent issues,’ and ‘analytical themes arising from the recurrence or patterning’ of issues, perspectives or concepts to sift and sort the dataset [55]. Additionally, the analytical framework was informed by the Theory of Systems Change [53] and the conditions for successful place-based governance, identified by Marsh and colleagues, namely ‘localised context’, ‘embedded learning’ and ‘reciprocal accountability’ [48]. Once agreement about the coding framework was reached, each code was assigned a brief definition to ensure congruity throughout its application to the remaining documents (third stage).

In the fourth stage, the codes were grouped into overarching thematic groups. Consistent with the framework method, we: 1. focused on ‘judgements about meaning, about the relevance and importance of issues, and about implicit connections between ideas’ ([55], p. 180); 2. developed charts for each thematic group so that the data could be ‘lifted’ from their original context and organised for a more streamlined review; and 3. kept the same order of Departments in all charts so that ‘comparisons could be made within or between cases’ ([55], p. 184). The chart headings were iteratively identified to capture the most significant characteristics of the thematic groups. Two authors (BK and AM) then systematically charted the coded data into the framework matrix, distilling and summarising the major themes related to ‘place-based’ throughout the dataset. In the fifth stage, two authors (BK and AM) engaged in data extraction and analysis and distilled the data that were both ‘typical’ and ‘exceptional’ [66]. Moving from the second to the fifth stage, we relied on a cyclical pattern instead of a linear one, and all findings were further validated through constant ‘member checking’ [67].

## Results and Discussion

In this paper, we combine the Results and Discussion sections to avoid repetition and redundancies and present the key arguments and interpretation of the results in an accessible and cohesive manner. Combining these sections in qualitative data analysis allows for a more cohesive presentation of findings due to the interpretive nature of qualitative analysis, the iterative process of coding and analysis, and the integration of findings and theoretical concepts.

### General characteristics of documents

**Fig. 1:**
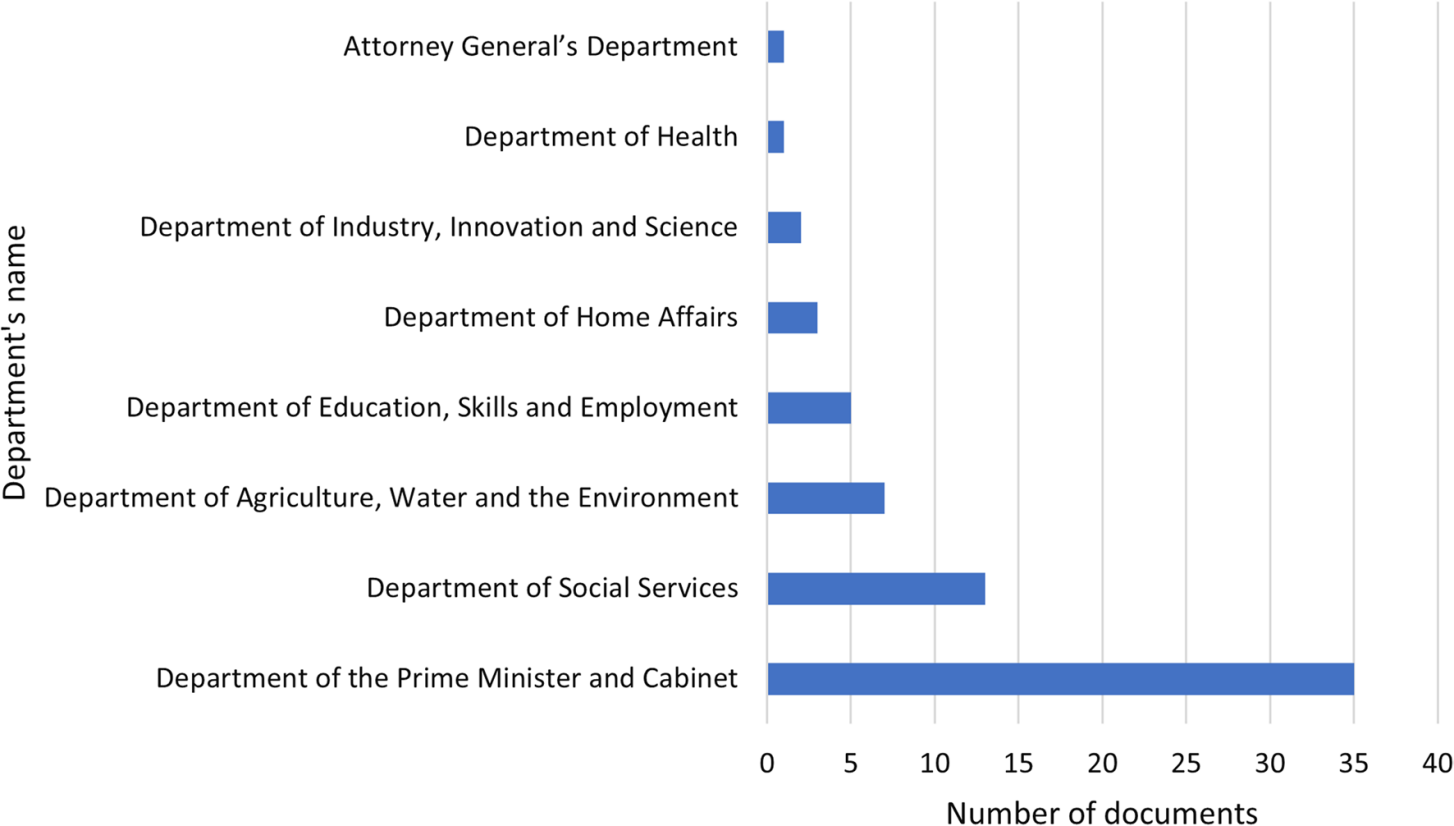
Number of documents by the departments

**Fig. 2:**
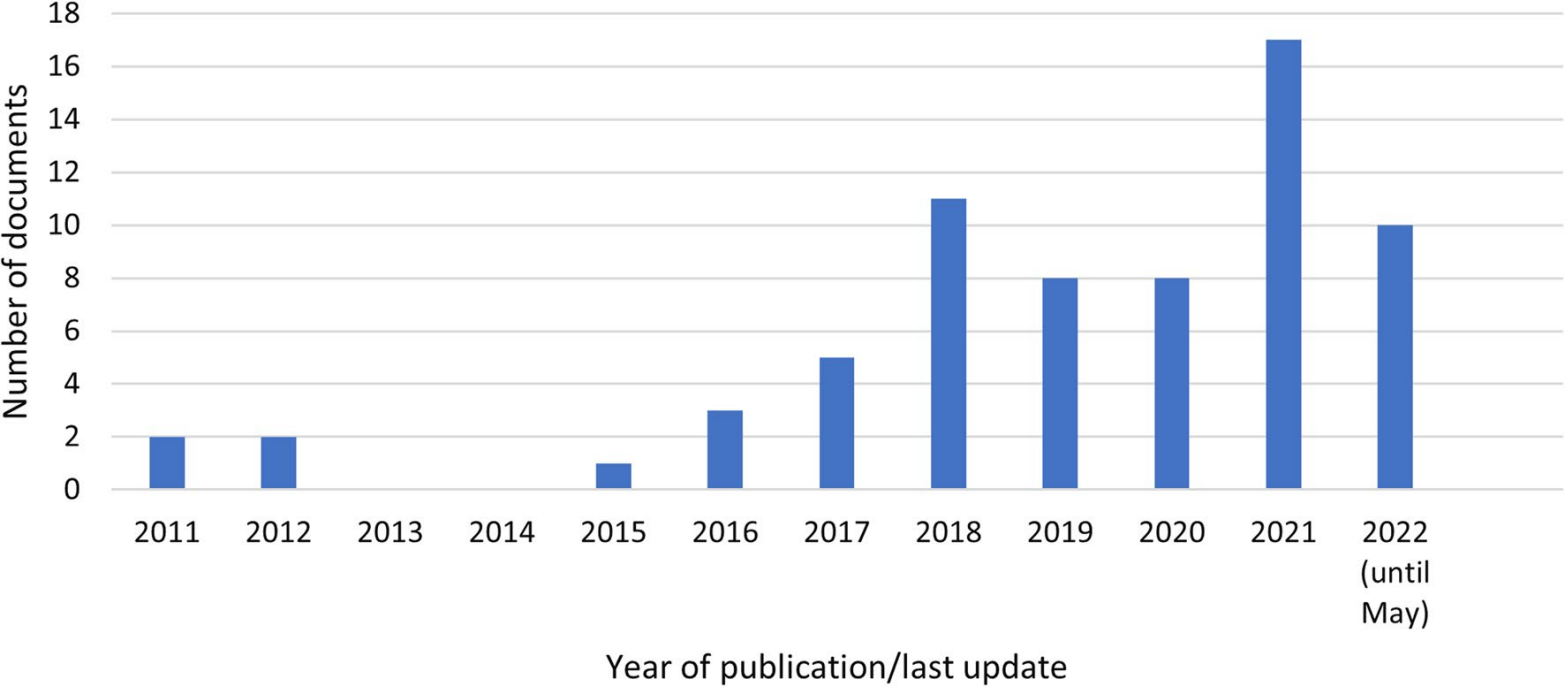
Number of documents by year

A total of 67 documents met the inclusion criteria; of these, we labelled 19 as primary and 48 as secondary. Most documents originated from the Department of the Prime Minister and Cabinet (*n* = 35; 52%; Fig. 1). We found that the number of documents that used the term ‘place-based’ increased after 2017 (Fig. 2), consistent with the increased use of the term in academic literature.^2^ The vast majority were published during or after 2018 (*n* = 54; 81%; Fig. 2), which may indicate increased interest in place-based approaches from ‘the top’, that is, from the Department of the Prime Minister and Cabinet, which is the central department of the Australian Government public service.

The primary documents (*n* = 19) originated from the following departments: the Department of Social Services (*n* = 7)[68–74]; the Department of the Prime Minister and Cabinet (*n* = 7) [59, 60, 75–79]; the Department of Home Affairs (*n* = 2) [80, 81]; the Department of Health^3^ (*n* = 1) [82]; the Department of Industry, Science, Energy and Resources^4^ (*n* = 1) [83]; and the Department of Education, Skills and Employment^5^ (*n* = 1) [84]. The vast majority of the primary documents (*n* = 17, 89%), were published during or after 2018. No documents were found for the following departments: the Department of Defence, the Department of Finance, the Department of Foreign Affairs and Trade, the Department of the Treasury, the Department of Veterans’ Affairs.

### Definitions, conceptualisations, and characteristics of ‘place-based approaches’

Place-based approaches have been challenged for their lack of ‘conceptual clarity and operational precision’ ([30], p. 562). Ensuring conceptual coherence across and within different public sectors, levels of government, and stakeholders involved in place-based approaches is considered important for the success of approaches relying on collaborative governance. Indeed, alignment within and across government was found to be an enabling factor for the take up of Health in All Policies [85].

Our analysis revealed that most documents (*n* = 59) did not define place-based approaches explicitly. Among those that did offer definitions (*n* = 8), the Department of Social Services authored the majority (*n* = 5). For the full list of definitions, see Additional file 1. The definitions provided by the Department of Social Services generally characterise place-based approaches as being: ‘collaborative’ [68, 70, 74, 86]; ‘long-term’ [68, 70, 74]; receptive to ‘complex problems’ [68, 70]; geographically defined [68, 73]; and focused on systemic (as opposed to programmatic) responses [70, 71, 86]. The definitions provided by the Department of Education, Skills and Employment and the Department of Health were less specific, describing place-based approaches as: ‘local solutions to issues in a specific location or region’ [84] and ‘policy, program or service approaches that recognise and respond to the characteristics of the community in which they operate’ [82], respectively. In the documents we analysed, ‘place-based’ was typically used to describe ‘place-based approaches’, with place-based initiatives, models and policies sometimes used synonymously.

While there was an absence of explicit definitions (except those described in Additional file 1), we found patterns in the characteristics attributed to or associated with place-based approaches, several of which are consistent with systems thinking perspectives. A ‘place-based approach’ was generally used to describe an effort that involved:

- collaboration between multiple stakeholders [59, 60, 68, 70, 71, 75, 79, 81, 82, 84, 86];
- including community^6^ in decision making [59, 68, 79, 81, 82, 84];
- responsiveness to community priorities/needs/issues [73, 76, 79, 82, 84];
- ‘understanding the place’ [59, 60] and valuing local knowledge [84];
- addressing complex issues [70, 84, 86] in a specific geographic location [68, 73, 84]; and
- alignment across existing programs and alignment between those involved in a place-based approach in the form of a shared vision [70, 74, 83].

In addition, we found that ‘place-based’ was applied to a range of services, contexts and problems, including ‘care’, ‘housing’, ‘early intervention’ and ‘partnership’ [82]; ‘service’ and ‘service system’ [69, 71, 82]; ‘network’ [69]; ‘event’ [84]; ‘solution’ [72, 75, 84]; ‘collaboration, ‘reform’, and ‘agenda’ [86]; ‘innovation ecosystem’ [83]; ‘narrative’ and ‘understanding’ [81]. In other cases, ‘place-based’ was used to describe initiatives, projects, events or services that may have been centred on a specific geographic location but did not appear to have other characteristics often attributed to place-based approaches.

These findings mirror the inconsistencies in the definitions and conceptualisations of place-based approaches in the academic and grey literature. Flexibility and pluralism in the government’s conceptualisation of place-based approaches could facilitate a more diverse take up of the concept, contributing to a growing but still fledgling field. Narrowing our conceptualisation of place-based too soon may hinder creativity [87]. On the other hand, a lack of clarity can also come with some costs, including confusion or misinterpretation contributing to ‘weak implementation’ [88], a lack of alignment across involved actors, and less effective policy communication due to inconsistent messaging. For public policy to create an enabling environment for place-based approaches to address complex problems and consistent with systems thinking perspectives, conceptualisations must be flexible enough to adapt to changing and emergent circumstances [89] while maintaining enough conceptual consistency to facilitate alignment across multiple sectors and settings.

Articulating the characteristics or ‘common attributes’ of place based approaches identified through this analysis and other literature could create an appropriate level of conceptual consistency. Rather than a prescriptive and narrow definition, these attributes could form an “ideal type” [30]. An ideal type describes key characteristics that can be applied in different contexts, to serve different populations and tackle different complex problems. Defining and communicating an ideal type is consistent with one of the analysed documents highlighting the potential for the Australian government to ‘play a greater role in coordinating and communicating' place-based policy ([83], p. 4).

Based on the findings above, we propose the following attributes or characteristics of place- based approaches, described as an ideal type.

*Place-based* *approaches* *are* *collaborative* *programs,* *interventions,* *or* *initiatives* *in* *which* *multiple* *stakeholders,* *united* *by* *a* *common* *vision,* *draw* *on* *the* *skills,* *knowledge* *and/or* *experience* *of* *local* *people* *to* *address* *complex* *issues* *within* *a* *specific* *geographic* *location.* *Recognising* *and* *leveraging* *the* *influence* *of* *‘place’* *on* *population* *outcomes,* *place-based* *approaches* *are* *context-dependent,* *responsive* *to* *the* *shifting* *needs* *and* *priorities* *of* *the* *places* *in* *which* *they* *are* *implemented* *and* *include* *‘people* *in* *place’* *and/or* *local* *organisations* *in* *decision-making.*

In proposing this ideal type, we intend to build understanding across the field as a step towards developing the federal government’s public policy framework, fostering an aligned approach to support place-based work across Australia [90].

Beyond these characteristics, our analysis revealed a number of commonalities in the problems, expected outcomes and populations that are the focus for place-based approaches.

### What are place-based approaches suitable to address and for whom?

Many policy documents present place-based approaches as well-suited to addressing complex problems, many of which are determinants of health. In the policy documents, persistent locational disadvantage and poverty are examples of the complex problems addressed through place-based approaches. For example, the Department of the Prime Minister and Cabinet mentions the ‘multidimensional’ [78] nature of locational disadvantage, and the ‘numerous and interrelated’ [76] problems that emerge as a result. Documents from the Department of Social Services highlight the immutable nature of disadvantage [68] and acknowledge that both ‘current policy settings and program interventions’ [70] and traditional ‘service-based program delivery’ are ineffective in the face of its ‘multiple and intersecting causes’ [86]. These discourses resonate with the general consensus in the literature that emphasises the potential of place-based approaches to address complex problems [91–93].

The expected outcomes of place-based approaches varied depending on the nature of the problem being addressed and the focus of the initiative. For instance, it was proposed that place-based approaches can contribute to the delivery of ‘tangible’ outcomes [60, 68, 70–72, 76, 86] that ranged from very broad, such as positive, significant, and sustainable population health and wellbeing outcomes to more specific ones such as improving networks for jobseekers [84] (see Table 1). In terms of determinants of health, as they are categorised in the Australian National Preventive Health Strategy 2021–2030 [5], place-based approaches were mainly framed as suitable to address socio-economic determinants of health.

**Table 1:** Expected outcomes identified in the documents categorised based on determinants of health

Determinant of health	Expected outcomes	References
Social (e.g., family situation, early childhood, housing, working conditions, social support and participation)	• Improving social outcomes	[68, 70, 71, 75, 76, 86]
	• Building healthy family environments	[69–72]
	• Fostering healthy child development (e.g. general wellbeing and family violence prevention)	[68–72]
	• Building social capital, community capacity and connections	[60, 69, 73, 86]
	• Increasing collaboration and trusting relationships between stakeholders	[83, 86]
	• Improving networks for jobseekers	[84]
Economic (e.g., education, employment, occupation, and income)	• Improving economic outcomes	[68, 75, 76, 79, 80, 82, 83]
	• Supporting education and learning for children and/or young people	[69–71, 80, 82]
	• Reducing poverty	[69, 70]
	• Creating business and employment opportunities	[71, 75, 80, 82–84]
Environmental, that is, natural (e.g. climate change) and built environment (e.g. urban design and transport)	• Improving environmental outcomes	[76]
	• Reducing risks from natural disasters	[81]
	• Driving regional and urban development	[83]
Structural (e.g. health care costs, service provision, systemic attitudes and practices, health literacy)	• Achieving ‘systemic change’ through ‘system-level reforms’	[70, 82, 86, 94]
	• Improving coordination, integration, accessibility, and relevance of services (e.g. primary health, allied health, social services…)	[68–71, 73, 75, 76, 82, 84]
	• Investing in early intervention and prevention	[60, 69, 70, 75]
	• Improving public service practices	[76, 78]
Cultural (e.g., connection to country and self-determination and leadership)	• Empowering and/or engaging ‘people on the ground’	[59, 60, 73, 75, 82, 86]
	• Increasing engagement and participation of Aboriginal and Torress Strait Islander people	[68, 71, 72, 75, 82]
Digital (e.g., telehealth, data, and digital platforms)	• Establishing digital platform to inform evidence-based service delivery	[69]
	• Improving access to and sharing of data	[73]
Commercial (e.g., corporate activities, supply chains)	• Driving innovation	[83]

In terms of target population groups, place-based approaches were broadly framed as an effective way to address the problems experienced by vulnerable or ‘at-risk’ cohorts experiencing significant, localised or ‘entrenched’ disadvantage [69, 71, 84]. The complex nature of disadvantage means that many cohorts targeted by place-based initiatives experience multiple and intersecting forms of disadvantage and vulnerability. When discussing place-based approaches, the documents tended to focus on specific attributes such as ethnicity or culture as antecedent to disadvantage without acknowledging the ways in which social and cultural constructs interact with and reinforce the marginalisation and discrimination that particular groups of people face [95]. The main target populations identified across the documents are listed in Table 2.

**Table 2:** Main target populations identified across the documents

Target population	References
‘Vulnerable cohorts’, that is, those who are experiencing significant disadvantage or are otherwise considered to be ‘at risk’	[69, 71, 84]
Aboriginal and Torres Strait Islander communities	[68, 70–72, 75, 82, 86]
Children, young people, and their families	[68, 71, 72, 75, 86]
Humanitarian entrants and other migrants	[71, 72, 80]
Job-seekers and people experiencing long-term unemployment	[75, 84]

Overall, expected outcomes were generally overly ambitious (e.g., achieving systemic change) and vaguely defined (e.g., improved social outcomes), while target population was broadly defined (e.g., vulnerable cohorts). Place-based approaches can be challenging to evaluate due to their complex and context-specific nature [92, 96, 97] and vague outcomes and target population groups further contribute to this challenge. Realistic outcomes should be more precisely defined and measured at both the system (e.g., alignment of services with the strengths and needs of the target population) and target population levels (e.g., education outcomes for children from low-income families).

### Government roles in place-based approaches

The most prominent roles occupied by the federal government were associated with: 1. the provision of funding [69–71, 75, 79]; 2. partnering with community [59, 60, 68, 70, 71, 75, 79, 81, 82, 84, 86]; and 3. the creation of a supportive policy environment [70, 75, 76, 78, 82–84]. Some other roles the government occupied included brokering relationships [84], which in one instance included building trust and relationships and investing in building the capacity of others [70], encouraging participation in communities of practice [83], and implementing place-based approaches [71].

#### Government-as-funder and Government-as-partner

Government funding plays a significant role in supporting the implementation and sustainability of place-based approaches [69–71, 75, 79]. However, much of the discourse around the government’s role as a funder was limited to *what* or *who* is funded, with *what* *amount* and for *how* *long*. Moreover, traditional government funding models were mentioned as a potential barrier to implementing place-based approaches, whereas flexible funding models were a potential enabler [86]. A more comprehensive and nuanced discussion about the government's role as a funder could be incorporated into future policy documents. This discussion could include considering how power dynamics and resource distribution influence decision-making, access to funding, and the equitable distribution of resources among different places or communities.

Although there was limited discussion about their role of funder, the government’s role as partner in place-based approaches was prevalent. In the document outlining the *Stronger* *Places,* *Stronger* *People* *Model*—a prominent Australian place-based collective impact initiative—the government is referred to as a ‘collaborative partner’ [70] with a ‘stewardship role in allowing and supporting communities’ to lead the initiative whilst ‘championing the need to work collaboratively’ ([70], p. 25). A document from the Department of the Prime Minister and Cabinet suggests that ‘genuine partnerships with a common aim can improve the lives of Australians’ ([78], p. 12). The importance of government partnerships with Aboriginal and Torres Strait Islander people that are driven by the strengths and needs of their communities was also prominent [60, 76, 82, 83]. For example, the *Aboriginal* *and* *Torres* *Strait* *Islander* *Health* *Plan* *2021–2031* outlines a commitment from all governments to work ‘in partnership with Aboriginal and Torres Strait Islander people to drive solutions’ ([82], p. 2) by affording Aboriginal Community-Controlled Health Organisations ‘a genuine leadership role in program and policy design, development and implementation across governments’ ([82], p. 24).

In some of the documents, there was a recognition that placing a community or target population at the centre of designing or delivering a policy or intervention through a partnership approach requires decision-making responsibilities to be devolved to some degree to the community level [92]. References to the devolution of power were prominent in the documents associated with the Department of Social Services. For example, shared decision-making was highlighted as one of three activity areas suggested for the National Centre for Collaboration [86], and was described as ‘devolving accountability, decision-making, funding and service delivery to the local level to facilitate strategically-targeted solutions tailored to community needs’ ([86], p. 4). However, the Department of Social Services also acknowledged that a ‘shift in power’ is required to support community-led decision-making [70]. This perspective is mirrored by broader discourses in the governance literature, which suggest that shifting 'direct control by ‘government’ to collaborative, multi-level ‘governance’ involving a range of actors across sectors’ ([51], p. 580) can play a key role in improved outcomes for people experiencing localised problems characterised by complexity [98]. The latter suggests that power be devolved to a collaborative infrastructure of stakeholders from across the system depending on the nature of the problem, such as community organisations, schools, businesses, police or local government, who will be more likely to have their hands on multiple levers to effect change.

Achieving the devolution of power to the community is complicated by the dual roles of partner and funder occupied by governments in place-based approaches. This duality produces a tension that is entangled with enduring socio-political norms that shape the way we conceive of government and community, respectively. The ‘government-as-partner’ role is often framed as an equal partnership between the government, community, and other involved stakeholders. It often implies both a ‘shared commitment’ and a ‘shared accountability for planning, decision making and results’ [74]. On the other hand, the ‘government-as-funder’ role demonstrates a natural power imbalance between the *partner* providing funding and the *partners* who are recipients of this funding. The unequal access to resources associated with such a role may challenge the concept of ‘shared power,’ and may indicate a lack of operational independence for the funded partner [99].

Tensions between the role of partner and funder are reflected in the critiques often levied at participatory governance mechanisms, in that they tend to ignore ‘structural, institutional and historically-determined inequalities in power between different partners’ and thus create ‘little change in power structures or dynamics’ ([51], 580). However, drawing on Giddens’ conceptualisation of power as the ‘transformative capacity’ to get things done or 'make a difference' [100], and acknowledging power is continuously exercised through our interactions with others, this natural power imbalance between the funder and the recipient of funding may not necessarily represent an unassailable barrier to a successful partnership. The nature of such imbalances may be addressed through building trust between involved partners [44, 101], careful consideration of risks, responsibilities, and power as it relates to both the government and other partners in place-based approaches [51], building capacity of all partners to engage in decision-making processes [47], enhancing transparency about expectations from partnerships, and creating a policy environment that will enable these processes [102].

#### Creating a supportive policy environment

In Australia, there has been a long history of advocacy for establishing supportive policy environments to facilitate the implementation and success of place-based approaches [90, 103–105]. Our analysis reveals that the government acknowledges the significance of this narrative and recognises its role in creating such supportive policy environments. Several government departments highlight the importance of supportive policy settings as a substantial government contribution to the effectiveness of place-based approaches. For example, the Department of Industry, Science, Energy and Resources highlights the role of both state and local governments in providing supportive policy settings to foster the growth of innovation precincts that adopt a place-based approach [83]. For the Department of Social Services, government-led ‘policy, funding and systems reform’ features as a fundamental component of the *Stronger* *Places,* *Stronger* *People* place-based collective impact initiative, and serves as one of its eight participation requirements for Commonwealth, state and territory governments ([70], p. 27). According to the *Stronger* *Places,* *Stronger* *People* theory of change, achieving system impact relies on improving ‘policies, practices, norms and service models… at the community and government levels’ and ensuring ‘aligned policy framing, investments and coherence of strategy’ ([70], p. 20). Yet despite its apparent centrality, the government’s commitment to policy reform is limited to an *exploration* of ‘ways to coordinate investment and align policies to support communities’ ([70], p. 27). Beyond this commitment to exploration, the *Stronger* *Places,* *Stronger* *People* model does not provide specific actions or strategies on behalf of government departments or partners seeking to substantiate policy reform. The need for an exploratory phase indicates that further research is needed into the design and implementation of novel policies, such as adaptive policies from systems thinking literature [89], that better support the successful implementation of place-based approaches.

The significance of establishing a supportive policy environment [70] was further evidenced in discussions about revising internal government practices to support place-based approaches better. Policy documents highlight a need to establish mechanisms to enable cross-departmental and cross-sectoral data sharing, recognising the importance of government systems that promote internal collaboration and information exchange [70, 76, 78, 84]. The Department of Prime Minister and Cabinet takes this concept a step further by committing to exploring ‘ways to encourage the application and broader adoption of place-based approaches across the public service’, to develop recommendations for ‘a more place-centred, transformational and joined-up delivery approach’ to public service delivery ([76], p. 18). This operationalisation of place-based approaches is noteworthy as it expands the understanding of *place* beyond the conventional geographical sense. Instead, it refers to the workplaces within the Australian entities that employ staff under the Public Service Act 1999 [106]. While the practical aspects of adopting place-based approaches within the public service are not elaborated upon in the policy document, this unconventional discourse suggests an evolution from the government’s traditional roles as funder and partner in place-based approaches, and indicates a recognition that to better support place-based approaches, the structures and machinations of government also need attention.

### Criteria for successful place-based governance

Given the significance of *good* *governance* for the success of place-based approaches, both in the policy documents and the broader literature, it is important to understand how the government perceives, communicates, and participates in governance structures related to place-based approaches. Scholars have regularly pointed to the tensions between the formal, institutional, and centralised service design of traditional *government* and the progressive development, discretion and decentralisation required for successful *governance* [49, 50, 107]. To surface these tensions in the policy documents, we turn to Marsh and colleagues’ three interconnected criteria for successful place-based governance—*localised* *context*, *embedded* *learning* and *reciprocal* *accountability* [48].

#### Localised context

Localised context highlights the need for place-based approaches to be flexible enough to cater to the unique needs of individuals and communities in place [48]. Across the policy documents, the importance of tailoring place-based approaches to the local context was emphasised. For example, The *National* *Aboriginal* *Torres* *Strait* *Islander* *Health* *Plan* *2021–2031*, developed by the Department of Health, emphasises the need for policy approaches that ‘foster an environment where adults feel empowered to determine their own health priorities’ ([82], p. 80). The government highlighted the importance of taking into account community priorities, needs, and issues [73, 76, 79, 82, 84], and identified a need to ‘understand the place’ [59, 60] and value local knowledge [84]. The policy documents also framed community involvement in the design, delivery, and decision-making processes as an integral component of successful place-based approaches [59, 68, 71, 79, 81, 82, 84].

#### Embedded learning

Successful place-based governance requires ‘continuous improvement and reciprocal learning’ that is ‘pragmatic, adaptive and experiential’ to be embedded at the core of place-based design ([48], p. 445). Such learning is also referred to in the systems thinking literature as ‘system action learning’ [108] and management literature as ‘adaptive management’ [109] and has some similarities with ‘dialogic learning’ [110]. It places attention on using evidence to inform future actions and improvement through learning cycles, that is, the iterative process of planning, implementing, evaluating, and adjusting based on the unique needs and characteristics of a particular community [108, 111, 112]. The collective impact design of the *Stronger* *Places,* *Stronger* *People* initiative emphasizes ‘data, shared measurement, evidence-informed decision-making, evaluation, and learning’ ([70], p. 8). Cycles of learning are embedded into this initiative and explicit support is provided for backbone organisations to build capacity in this area. Moreover, *Stronger* *Places* *Stronger* *People* extends the importance of learning ‘by doing and adapting’ to the government and encourages active participation ‘in learning processes and evaluation, including critically examining the role and contributions of governments in enabling successful implementation.’ ([70], p. 26).

Apart from *Stronger* *Places* *Stronger* *People,* there was limited emphasis on embedded learning beyond the general encouragement to share best practices, data, learnings, and information [72, 76, 83]. Meaningfully embedding learning cycles into the design and delivery of place-based approaches can contribute to addressing the challenges associated with disentangling the effects of place-based approaches through evaluation [92, 96, 97]. Evaluation approaches and methods that support rapid feedback and learning cycles, including real-time evaluation, developmental evaluation, rapid-feedback evaluation, rapid assessment participatory rural appraisal [113], would be well-suited to the context-sensitive nature of place-based approaches. These evaluation approaches share ‘a similar set of techniques for putting trustworthy, actionable information in the hands of decision makers at critical moments’ ([113], p. 152), and focus primarily on learning and improvement rather than upwards accountability [114]. A learning and improvement focus is particularly important when attempting to alter systems, which can respond in unpredictable ways and with unintended consequences.

Efforts to systematically embed adaptive and experimental learning across all place-based programs may benefit from a policy environment that supports individual and organisational level capacity development. This could be achieved in several ways, such as through the implementation of monitoring mechanisms and systems that enable data sharing across departments and sectors [70, 76, 78, 84] as well as between partners involved in place-based programs. The Department of Social Services outlined the importance of creating ‘data sharing arrangements’ and building community capacity to access and utilise the data [68] by developing the focus areas for the National Centre for Place-Based Collaboration (currently under establishment). Additionally, public policy could support staff training for skill development in approaches such as developmental evaluation and support organisational leaders to embed ongoing learning in organisational policies, culture and systems.

Furthermore, data and reporting requirements must represent more than a ‘tick-box exercise in measuring easily quantified outputs,’ but instead are ‘envisaged as a conversation where review leads to continual improvement’ ([48], p. 444-445). In the case of *Communities* *for* *Children* *Facilitating* *Partners*, client data and service delivery information must be reported via the Department of Social Services’ Data Exchange. Reporting requirements mandate reporting centrally determined client and community outcomes via a standardised framework that uses a simple five-point rating scale. Qualitative outcomes data is not requested as part of the data exchange. One document indicates that the government is ‘aware that many service providers have expressed concerns about performance being assessed solely on client outcomes data reported through the Data Exchange’ [115], with concerns raised over the lack of qualitative data captured. While the accountability and reporting regime of the Data Exchange may be well-suited to some contexts, its use of centrally determined outcomes and focus on ‘best-practice’ suggest a ‘one-size-fits-all’ approach, which may create tensions with the place-based approach of *Communities* *for* *Children* *Facilitating* *Partners,* particularly concerning attempts to be sensitive to the local context [48].

#### Reciprocal accountability

Reciprocal accountability entails ‘a justification of local results against local targets set in the context of priorities determined by the centre’ ([48], p. 445). While some documents recognised the necessity of ‘devolving accountability’ [70, 73], ‘sharing accountability’ [86] or ‘moving accountability to the local level’ [74], there was an overall lack of references to clear and effective governance mechanisms to support reciprocal accountability in place-based approaches. Indeed, despite the centrality of localised context and community involvement, devolving accountability remains one of the most challenging goals of place-based approaches [32]. This sentiment is reflected in one document that suggests resistance to the uptake of programs by Indigenous families is in part informed by ‘a sense that governments may have sometimes been unwilling to devolve responsibility or partner with communities to determine solutions’ ([68], p. 26). Indeed, a lack of ‘accountability systems that are sophisticated enough to allow for the level of local differentiation required’ ([48], p. 445), indicates a need for further research in this area.

In summary, the analysis showed *localised* *context* was emphasised across the vast majority of the policy documents, *learning* seems to be systematically embedded into only one of the largest place-based approaches, and there was an overall lack of reference to governance mechanisms that support *reciprocal* *accountability*. The vertical integration of policy from government to community, outlined in a dominant focus on *localised* *context*, is warranted. However, less attention has been paid to multi-sectoral integration across government. This is reflected in the policy documents we reviewed in this study. This omission is problematic because we know that many of the complex problems communities face require action across sectors. Health in All Policies, as a framework designed to create alignment across government, may provide some guidance here [2, 85, 116].

### Implications for public policy

Based on our findings, the Australian government’s perspective of their involvement in place-based approaches involves a negotiation of both the function of *government* (i.e. the top-down processes which operate at the federal level to ‘maintain pubic order and facilitate collective action’ ([49], p. 15) and the practice of place-based *governance* (i.e. the bottom-up processes which allow for and encourage local decision making and accountability). Many of the policy documents we analysed acknowledged the importance of the bottom-up, community-driven development, implementation, and evaluation of place-based approaches. This was particularly evident in the emphasis placed on localised context as a necessary component of place-based approaches. On the other hand, the government’s perspective of their top-down involvement was more subtle, indicated predominantly by their financial commitment to place-based approaches and through acknowledging the need for supportive policy environments.

Evidence suggests hybrid models that integrate both top-down and bottom-up involvement tend to encourage more successful forms of governance [117–120], which is an approach the Australian federal government is already pursuing. However, given the disproportionate emphasis on the bottom-up approach across the policy documents, there is a danger the government’s interest and/or involvement in the development and sustainability of place-based approaches will diminish [44, 105] and leave communities already experiencing disadvantage with a "burden" to develop, monitor, implement and evaluate place-based approaches that is disproportionate to their capacities. As such, we suggest that governments engaged in place-based approaches should work towards a more balanced hybrid approach to place-based approaches that maintains the central functions of government while allowing for successful place-based governance. In addition, attention needs to be paid to cross-sectoral alignment across government, drawing on insights gained from Health in All Policies as well as systems thinking literature (e.g., see [121]), to ensure conceptual consistency both horizontally across government and vertically between government and communities. A well-balanced hybrid approach could contribute to establishing conceptual clarity and coordinated communication related to place-based approaches, reducing the tension between the government’s partner and funder roles, and strengthening their government’s top-down role as the creator of a supportive policy environment.

Based on our findings, we outline key suggestions for public policy that could contribute to adoption of a more balanced approach:

- *Promoting* *consistency* *in* *conceptualisation* *of* *‘place-based’* *within* *and* *across* *departments* *and* *sectors* *to* *support* *place-based* *initiatives* *across* *Australia*. One way to achieve consistency is through a balanced approach in the conceptualisation of place-based approaches that builds of the ideal type described earlier. Such consistency would assist with developing a national public policy framework for place-based approaches, whilst pluralism can be retained to foreground the differences between place-based approaches (e.g., different target groups, expected outcomes, specific characteristics of a place).
- *Employing* *an* *active* *role* *in* *trust* *building*: A ‘lack of trust in national institutions’ [77] was identified as a barrier to successful place-based approaches, yet only one of the place-based approaches featured in the policy documents emphasised trust and relationship-building as a necessary role of government [70]. Absence of trust is a major issue for public authorities and other institutions worldwide [122], and in Australia, many people see government as ‘a dividing force in society’ that is not able to ‘solve societal problems’ [123]. Ways of working that include many partners collaborating to solve complex issues, like joined-up government and place-based approaches, can create high expectations among involved partners [11, 44, 118]. Despite a mutual appreciation for collaboration and a ‘joined-up culture', trust may be eroded and 'reform fatigue' [88] can occur when expectations are not met. Building trusting relationships has been identified as a key strategy for improving joined-up-culture  [88], and is also an important enabler of successful community engagement in place-based approaches [101] and one of the core mechanisms for scaling up complex interventions [124] and enabling systems change [125]. Therefore, the government should consider adopting the active role of a broker that builds trusting relationships across all its place-based programs and allowing for time and funding that supports relationship development. For example, in the Victorian place-based Community Revitalisation initiative, a team of state government public servants undertook a dual role of a community partner and intermediary between the community and the government [126]. They adopted a ‘learnings-orientated’ approach using a range of reflective practices [126]. The longer funding cycle that supported relationship development and trust-building ‘helped to build trust between government and sites by breaking down traditional power dynamics and demonstrating that government is willing to listen’ ([126], p. 50).
- *Advancing* *the* *creation* *of* *a* *supportive* *policy* *environment*: Efforts to create a supportive policy environment for place-based approaches would benefit from the development and communication of policy providing specific guidance on the types of governance arrangements that best support local decision-making and reciprocal accountability and the types of systems and structures that enable communities to drive and/or implement place-based approaches. Additionally, the government’s interest in revising internal government systems and practices to establish an enabling policy environment to better support place-based approaches could be further expanded. For example, the State Government of Victoria developed a framework for place-based approaches to ‘start a conversation about how government can better support place-based approaches’ ([102], p. 3). The development of a national public policy framework for place-based approaches could be adopted at the federal level [90] as a step towards creating a consistent approach to support place-based approaches in Australia. Finally, to create a supportive policy environment for place-based approaches, the government could improve efforts for horizontal integration and alignment across sectors and learn from successful implementation of Health in All Policies [85, 116, 127, 128]. This could include actively forming cross-sectoral relationships to develop policies that are aligned across sectors and can more efficiently support the funding, design, governance, and implementation of place-based approaches. Creating a supportive policy environment and establishing policies that actively promote and facilitate cooperation across sectors could lead to better population health outcomes through several mechanisms such as resource optimisation, data sharing, alignment of services, and development of place-based initiatives and/or comprehensive interventions. We suggest that systems thinking methods, such as network analysis and mapping of the government system to explore current cross-sectoral relationships, could be used to inform and/or improve cross-sectoral collaboration, alignment and action.
- *Embedding* *learning* *across* *place-based* *approaches:* As an integral criterion for successful place-based governance, continuous, adaptive learning should be systematically embedded in design and delivery of place-based approaches. Governments could leverage their top-down role to contribute to a more systematic implementation of learning across all place-based programs, through for example implementation monitoring mechanisms and data sharing arrangements across and within departments and sectors as well as with external partners (e.g., community members, researchers, practitioners). Additionally, governments could consider developing and implementing so called “adaptive policies” given their inherent compatibility with characteristics of place-based approaches and systems thinking perspectives. Such policies are more flexible than static policies and may be are better suited to support continuous learning and adaptation to unanticipated shifts and changes [89]. Furthermore, it is advisable for future policy documents to provide clearer and more attainable expected outcomes of place-based approaches as ambiguously defined or excessively ambitious outcomes can create additional difficulties when evaluating place-based approaches.

### Implications for future research

To develop the research agenda on place-based approaches we recommend future research consider supporting policy and practice by building on existing research exploring: 1. types of governance arrangements that are most effective to support place-best approaches [44, 48, 104]; 2. flexible funding models and their implementation and efficiency in practice [129–131]; 3. characteristics of a public policy environment that supports place-based approaches [90]; and 4. types of accountability systems that can support successful place-based governance [48]. Additionally, to further build the evidence base around place-based approaches, especially related to public policy and the role of governments, future research could analyse public policies of other governments related to place-based approaches using and/or refining our methodological approach. Future research should consider analysing Australian state and local-level public policies and public policies (on any level) of other countries engaged in the development, implementation and/or evaluation of place-based approaches. To advance the application of research on place-based approaches into practice and policy, researchers, practitioners, policy-makers and others engaged in development, implementation and/or evaluation of place-based approaches could use, provide feedback and/or further refine our ideal–typical suggested conceptualisation of place-based approaches. Finally, since there has been limited rigorous examination of public policy relating to place-based approaches through a complexity (or systems thinking) lens, a more in-depth analysis is warranted. This could, for example, include a comprehensive exploration of interactions and relationships between different government sectors and other components of the system such as community organisations and local businesses.

### Strengths and limitations

The main strengths of this article are: 1. the employment of an inclusive search strategy and broad eligibility criteria, which enabled us to find, review and analyse a broad range of policy documents; 2. the eligibility assessment of policy documents, data extraction, and data analysis conducted independently by two authors, reducing the likelihood of human error; 3. a novel conceptualisation of place-based approaches based on the findings; 4. contributing empirical data to theoretical constructs developed by Marsh and colleagues; and 5. the first rigorous review and analysis of public policy related to place-based approaches (to the authors’ knowledge).

The paper also has some limitations. Even though we used rigorous and well-established methodology to locate and analyse the policy documents, we are cognisant that ‘analysis of the policy text is not a simple and straightforward activity’ ([132], p.12) and that the analysis of policy texts always leaves room for (mis)interpretation. In this paper, we primarily focused on ‘what is clearly and openly articulated’, consequently identifying ‘the ‘silences’ (what is not stated)’ was beyond the scope of this work ([132], p. 12). Additionally, the scope of this research was limited to analysis of federal-level policies in the Australian context. Finally, the major federal place-based approaches already underway sit within the Department of Social Services (e.g., Communities for Children, Stronger Places Stronger People), and this is one reason for their overrepresentation in the dataset. We thus suggest interpreting our findings with these limitations in mind.

## Conclusions

Many of the policy documents lacked a specific definition of a ‘place-based approach’ but several common characteristics of place-based approaches were identified such as collaboration and alignment between various stakeholders, including the community in decision making, responsiveness to community needs, and suitability of place-based approaches to address complex problems in a specific geographic location and socio-economic determinants of health. We identified three roles of the government in place-based approaches, that is, a funder, a partner, and the creator of a supportive policy environment. From the three criteria for successful place-based governance, *localised* *context* was the most represented across the policy documents and *reciprocal* *accountability* the least [48].

Overall, the Australian government’s perspective of their involvement in place-based approaches included a negotiation of the function of government, that is, the top-down processes and the practice of place-based governance, that is, the bottom-up processes. However, there was a disproportionate emphasis on the bottom-up approach across the policy documents, which poses a danger that the government’s interest in place-based approaches could diminish [44, 105] and leave communities experiencing disadvantage with a ‘burden’ to engage in place-based approaches that is disproportionate to their capacities. Governments engaged in place-based approaches should work towards a more balanced hybrid approach to place-based approaches that maintains the central functions of government while allowing for successful place-based governance. Key suggestions to achieve this include: 1. promoting consistency in conceptualisation of ‘place-based’; employing an active role in trust building; 2: advancing the creation of a supportive policy environment; and 3. embedding learning across place-based approaches.

### Supplementary Information

**Additional file 1:** Definitions of place-based approaches identified in policy documents.

## Data Availability

The data that support the findings of this paper are available from the corresponding author upon reasonable request.

## Footnotes

1 On 1 July 2022: the Department of Agriculture, Water and the Environment was superseded by the Department of Agriculture, Fisheries and Forestry and the Department of Climate Change, Energy, the Environment and Water; the Department of Health was superseded by the Department of Health and Aged Care; the Department of Infrastructure, Transport, Regional Development and Communications became the Department of Infrastructure, Transport, Regional Development, Communications and the Arts. The Department of Industry, Science, Energy and Resources was superseded the Department of Industry, Innovation and Science on 1 February 2020. On 1 July 2022, it was superseded by the Department of Industry, Science and Resources and the Department of Climate Change, Energy, the Environment. The Department of Education, Skills and Employment was dissolved on 29 May 2019. It was superseded by the Department of Employment, Skills, Small and Family Business, which was dissolved on 1 February 2020 and superseded by the Department of Education, Skills and Employment and the Department of Industry, Science, Energy and Resources. The Department of Education, Skills and Employment was superseded by the Department of Education and the Department of Employment and Workplace Relations.

2 A brief search (with no limit to document type, subject area or year of publication) was conducted in Scopus using a broad search syntax: TITLE((place-based) OR (place based)). The search yielded 3091 documents, of which 1340 (43%) were published between 2018 and 2022. The search was performed on 4 August 2022.

3 This department was superseded by the Department of Health and Aged Care, on 1 July 2022.

4 This department was superseded by the Department of Industry, Science and Resources and the Department of Climate Change, Energy, the Environment, on 1 July 2022.

5 This department existed from February 2020 to 1 July 2022, and it was superseded by the Department of Education and Department of Employment and Workplace Relations.

6 It is important to note that ‘community’ is conceptualised in a range of ways throughout the documents. For example, when the Department of Social Services refers to community engagement, it generally means citizenry, whereas community partnerships are mostly with organisations and service providers. On the other hand, for the Department of Industry, Science, Energy and Resources, community generally referred to researchers, educators and people from specific industries in a local area. Different terms that were used interchangeably with community were: Australians, civic, public, citizens.
