# Supplementary material for: Government, governance, and place-based approaches: lessons from and for public policy
Source: Health Res Policy Syst. 2023 Nov 29;21:126. doi: 10.1186/s12961-023-01074-7 (PMC10685506; doi:10.1186/s12961-023-01074-7)
Supplement: Supplementary file 1 — Additional file 1: Definitions of place-based approaches identified in policy documents. [file 12961_2023_1074_MOESM1_ESM.docx]

Additional file 1 - Definitions of place-based approaches identified in policy documents

| **Department** | **Year** | **Definition of place-based approach** |
| --- | --- | --- |
| Department of Education, Skills and Employment | 2018 | ‘Place-based approaches are local solutions to issues in a specific region or location’ (1). |
| Department of Premier and Cabinet | 2018 | ‘A place-based approach recognises the impact of ‘place’ on individuals’ experiences and outcomes and incorporates this recognition into strategies to improve social, economic and environmental outcomes’ (2). |
| Department of Social Services | 2018 | ‘Place-based approaches are generally long-term responses to complex problems delivered in a defined geographic location’ (3). |
| Department of Social Services | 2019 | ‘Collective impact is a community led movement to achieve large-scale change, informed by broad and inclusive community and stakeholder engagement which provides a framework and resources to address poverty and disadvantage. It is informed by a shared vision and locally developed strategy and plan of action. It is a way of working with an emphasis on data, shared measurement, evidence-informed decision making, evaluation, and learning’ (4). |
| Department of Health | 2021 | ‘Place-based approach refers to policy, program and service approaches that recognise and respond to the characteristics of the community in which they operate’ (5). |
| Department of Social Services | 2022 | ‘[Place-based approaches] build on the local leadership to empower a locally-led agenda’ (6).  ‘Place-based approaches are collaborative, long-term approaches to building thriving communities, delivered in a geographic location’ (6). |
| Department of Social Services | 2022 | ‘Place-based initiatives bring the community together to improve the conditions in which families and communities live, work, and raise children, using local knowledge and skills’ (7) |
| Department of Social Services | 2022 | ‘[Place-based approaches involve] the shared commitment to a local strategy by communities, governments, service providers and investors, which shared accountability for planning, decision-making and results’ (8) |

# **References**

1. DESE. The next generation of employment services: Discussion paper. the Department of Education, Skills and Employment: Commonwealth of Australia; 2018.

2. DPMC. Australia’s Second Open Government National Action Plan. Open Government Partnership Australia: Department of Prime Minister and Cabinet: Commonwealth of Australia; 2018.

3. DSS. Stronger Outcomes for Families - Background Paper June 2018. Department of Social Services: Commonwealth of Australia; 2018.

4. DSS. Stronger Places, Stronger People Model. Department of Social Services: Commonwealth of Australia; 2019.

5. DOH. National Aboriginal and Torres Strait Islander Health Plan 2021-2031 [Internet]. Department of Health: Commonwealth of Australia; 2021. Available from: https://www.health.gov.au/resources/publications/national-aboriginal-and-torres-strait-islander-health-plan-2021-2031

6. DSS. National Centre for Collaboration Working Group - Outline of Requirements for a Grant for Foundational Work to Establish a National Centre for Place-Based Collaboration (Nexus Centre) [Internet]. Department of Social Services: Commonwealth of Australia; 2022. Available from: https://www.dss.gov.au/sites/default/files/documents/03_2022/d22-50399-discussion-paper-consultation-requirements-national-centre-february-2022-accessible.pdf

7. DSS. Supporting Communities - Budget 2022-2023. Department of Social Services: Commonwealth of Australia; 2022.

8. DSS. National Centre for Place-Based Collaboration (Nexus Centre) (webpage) [Internet]. Department of Social Services: Commonwealth of Australia; 2022. Available from: https://www.dss.gov.au/place-based-collaboration
